# Relative Amino Acid Composition Signatures of Organisms and Environments

**DOI:** 10.1371/journal.pone.0077319

**Published:** 2013-10-25

**Authors:** Alexandra Moura, Michael A. Savageau, Rui Alves

**Affiliations:** 1 Department of Basic Medical Sciences and Institute of Biomedical Research, University of Lleida, Lleida, Spain; 2 Department of Biology & CESAM, University of Aveiro, Aveiro, Portugal; 3 Department of Biomedical Engineering, University of California Davis, Davis, California, United States of America; The Centre for Research and Technology, Greece

## Abstract

**Background:**

Identifying organism-environment interactions at the molecular level is crucial to understanding how organisms adapt to and change the chemical and molecular landscape of their habitats. In this work we investigated whether relative amino acid compositions could be used as a molecular signature of an environment and whether such a signature could also be observed at the level of the cellular amino acid composition of the microorganisms that inhabit that environment.

**Methodologies/Principal Findings:**

To address these questions we collected and analyzed environmental amino acid determinations from the literature, and estimated from complete genomic sequences the global relative amino acid abundances of organisms that are cognate to the different types of environment. Environmental relative amino acid abundances clustered into broad groups (ocean waters, host-associated environments, grass land environments, sandy soils and sediments, and forest soils), indicating the presence of amino acid signatures specific for each environment. These signatures correlate to those found in organisms. Nevertheless, relative amino acid abundance of organisms was more influenced by GC content than habitat or phylogeny.

**Conclusions:**

Our results suggest that relative amino acid composition can be used as a signature of an environment. In addition, we observed that the relative amino acid composition of organisms is not highly determined by environment, reinforcing previous studies that find GC content to be the major factor correlating to amino acid composition in living organisms.

## Introduction

As early and in the 1930s, Alfred Redfield analyzed the oceanic ratios of carbon, nitrogen and phosphorus to find that they were approximately constant at 106C:16N:1P and similar to those observed in the organisms living in those ecosystems [1]. Later, Redfield suggested that this was a consequence of organisms maintaining the environmental abundance of the major chemical elements at homeostatic values closer to those in protoplasm [2]. Measurements of the Redfield ratio in other environments suggest that, even though they vary slightly, they are approximately constant for a given type of environment [3], [4].

Recent work also reveals that the Redfield ratios in individual organisms and clades deviates from the global values and is dependent on phylogeny, geochemical constraints, and nutrient availability [4]–[8]. In fact, long term deficits of a given environmental chemical nutrient can be a driving force for evolutionary changes in the composition of the enzymes that fix that nutrient. Such changes in the enzyme's composition usually lead to a decrease in the frequency of amino acids that contain large amounts of the limiting environmental nutrient [9]. At the molecular level, such biases are also observed and enzymes that synthesize specific amino acids, when they are absent from the environment, contain low relative amounts of their cognate amino acids [10].

Taken together, the above observations raise the following questions:

- Does environmental relative amino acid abundance (eRAAA) of the 20 naturally occurring protein L-α-amino acids have ratios that are analogous to the Redfield ratios for chemical elements?
- If so, are such ratios as widespread as those for C:N:P?
- Is cellular relative amino acid abundance (cRAAA) of each organism approximately the same as its habitat eRAAA, suggesting an efficient utilization of free amino acids by the microorganisms in a given community? If not, does each organism have distinctive cRAAA, suggesting that eRAAA is a complex function of the dynamics of amino acid production and turnover by microbial communities inhabiting such environments?
- Finally, could the environment feedback onto the organisms and contribute to the evolution of their whole cell amino acid composition in specific environments, as it appears to do for the nucleotide composition of genomes [11]?

To answer the first two questions we compiled complete amino acid measurements from different environments and compared them. We found that indeed there are specific signatures for eRAAA and that these signatures are broadly similar within each type of environment.

Answering the last two questions required an additional estimation of the cellular relative amino acid composition (cRAAA) of the different species inhabiting the various environments. Given that single-cell organisms represent the vast majority of biomass in aquatic and terrestrial environments [12], to estimate such cRAAA we used the predicted proteome composition of prokaryotes and unicellular eukaryotes with fully sequenced genomes. Our results showed that the population in any given environment is heterogeneous with respect to their cRAAA, suggesting that organisms evolved the ability to differentiate their cRAAA from that of their environment through the regulation of their amino acid biosynthesis and utilization pathways.

## Materials and Methods

### Data collection and classification

We collected more than 100 different environmental determinations of amino-acid natural abundances. Out of these, we only retained those measurements that simultaneously determined at least 16 out of the 20 L-α-amino acids (n = 69, see **Table** **S1** and references therein), covering a wide spectrum of habitats, including water bodies, land masses and intestinal environments. Determinations of Asp/Asn and Glu/Gln were considered together for the analysis, because environmental measurements did not distinguish between the two amino acids in the pairs. Environments were classified in terms of aquatic (ocean and freshwater environments), terrestrial and host-associated environments.

Completely sequenced genomes and predicted protein sequences of unicellular organisms were obtained from the KEGG and NCBI databases [13], [14]. Similar strains were discarded from the analysis to reduce phylogenetic bias. Genomes used in further analyses (*n* = 1086) included 961 Bacteria, 72 Archaea and 53 Eukarya (**Table** **S2**).

Organisms were classified in terms of habitat (aquatic, terrestrial, versatile, specialized and host-associated) based on information retrieved from the Integrated Microbial Genomes [15], Genomes Online [16] and NCBI Genome Project [17] databases and from the primary literature.

### Calculation of genome and proteome properties

Using locally developed PERL scripts, we estimated the following properties for each organism from completely sequenced and fully annotated genomes: GC content, base pair composition of genes, codon usage and absolute amino acid abundance. These were important to control for the influence of non-environmental factors on protein amino acid composition.

### Estimation of cellular amino acid content

Since it was not feasible to obtain experimental determinations of the cRAAA for all the organisms used in this work, we estimated cRAAA from an organism's predicted protein abundances assuming that cells grow without nutrient restrictions. In such conditions, the level of expression of the different proteins in the genome can be estimated with respect to that of abundant ribosomal proteins [18]–[22].

In this way, the average amino acid composition of each organism was calculated using locally developed PERL scripts by weighting the abundance of each protein with respect to the ribosomal proteins, whose abundance was set to be maximal. Two different metric functions were used to weight protein abundance, CAI and a δ index.

CAI defines translationally optimal codons [23]. To calculate it, we normalized the data using the relative adaptiveness (*w_c,a_*), as previously described [22]. This adaptiveness was calculated for ribosomal proteins, in which the frequency of each synonymous codons (*n_c,a_*) was normalized by the frequency of the most frequent codon (being *C_a_* the set of synonymous codons used by amino acid *a*):
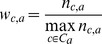



Thus, the codon usage of each coding sequence was represented by a vector of length 59 (stop codons and amino acids with only one codon were discarded). CAI was then computed for each gene by summing over the codon usage vector (rather than over the length) [22]:
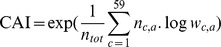



Here, *n_tot_* is the total number of codons in the gene.

To compute the δ index, first we measured the Euclidean distance (*γ_p_*) between the codon usage of protein *p* (CU_c,protein p_ representing the average relative usage of codon *c* in protein *p*) and the average codon usage of ribosomal proteins (CU_c,rib_):
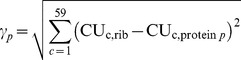



Then, we defined δ as an independent weighting function for gene expression as follows:
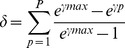



Here, γ_max_ is the maximum Euclidean distance in a given genome.

CAI and δ range between 0 and 1 for any given gene, with higher values indicating genes that are more expressed, thus having higher contribution to organism's amino acid content.

The cRAAA of each organism was computed as a vector of 20 amino acids, in which the cellular relative abundance of each amino acid (*cRAAA_aai_*) was calculated as follows:
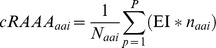



Here, *n_aai_* is the frequency of amino acid *i* in each protein, EI is the predicted expression index (CAI or δ) and *N_aai_* is the total count of that amino acid in the organism's proteome.

As a control, predictions were compared with experimentally-determined amino abundances in *Bacillus subtilis, Escherichia coli* and *Staphylococcus aureus* retrieved from the literature [24]–[26].

### Statistical analyses

Multi-dimensional matrixes of cRAAA and eRAAA were generated in which each column represented the relative content of each amino acid and each row represented each organism or environmental measure, respectively.

Principal Component Analysis (PCA) and Hierarchical Clustering were carried out to analyze the segregation of environments and organisms as a function of eRAAA and cRAAA, respectively.

To understand if environmental factors significantly contributed to shape global amino acid composition of organism, we needed to control for the factors which are already known to explain that composition: genomic GC content and phylogeny [11]. In addition we considered the main habitat of each organism. Finally, we created a generalized linear model of amino acid composition as a function of these three factors in order to estimate the effect of each factor on that composition:
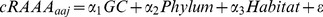



In this equation *cRAAA _aaj_* is the cRAAA of amino acid j, α_1_, α_2_, and α_3_ are linear coefficients and ε is a noise term [27]. The variables *Phylum* and *Habitat* were treated as discrete categorical data and given integer values (e.g. Crenarchaeota → 1, Euryarchaeota → 2, Korarchaeota → 3, Nanoarchaeota → 4, Acidobacteria → 5, Actinobacteria → 6, etc.; and Aquatic → 1, Terrestrial → 2, Versatile → 3, Specialized → 4, Host-associated → 5, Gut → 6, respectively). We created independent models for each amino acid and estimated the coefficients and their significance using ANOVA analysis [28].

Linear and Rank Correlation analyses between environmental and cellular amino acid compositions were performed based on Pearson's and Spearman's correlation coefficients, respectively. Statistical significance of the correlation coefficients was calculated using a t-test [28].

All statistical analyses were done using Wolfram Mathematica 8.0 (Wolfram Research, Inc., USA).

## Results

### Environments share amino acid signatures

**Table 1** shows the average environmental relative amino acid abundances (eRAAA) obtained based on the experimentally determined amino acids measurements collected from the literature (**Table** **S1** and references therein). Overall, eRAAA ranged between 0.2% and 16%, with Gly, Ser, Ala, Asn+Asp and Gln+Glu being the most abundant amino acids (mean >10%).

**Table 1:** Average environmental relative amino acid abundance (eRAAA) across habitats calculated from the literature.

Amino	Aquatic	Terrestrial	Host-associated	All environments
acid	(mean ±SD)	(mean ±SD)	(mean ±SD)	(mean ±SD)
**Ala**	0.1167 ±0.05315	0.1144 ±0.01689	0.0602 ±0.00516	0.1113 ±0.04662
**Arg**	0.0378 ±0.03249	0.0459 ±0.03462	0.0504 ±0.01340	0.0409 ±0.03191
**Asn+Asp**	0.0896 ±0.04352	0.1435 ±0.04755	0.0618 ±0.00557	0.1009 ±0.04980
**Cys**	0.0005 ±0.00265	0.0015 ±0.00323	0.0351 ±0.00332	0.0037 ±0.01004
**Glu+Gln**	0.0883 ±0.04270	0.1696 ±0.13768	0.2039 ±0.01316	0.1187 ±0.08787
**Gly**	0.2002 ±0.11806	0.0951 ±0.04960	0.0781 ±0.00580	0.1633 ±0.11176
**His**	0.0134 ±0.02044	0.0328 ±0.03000	0.0199 ±0.00191	0.0189 ±0.02374
**Ile**	0.0375 ±0.02426	0.0266 ±0.00944	0.0349 ±0.00138	0.0345 ±0.02075
**Leu**	0.0604 ±0.02298	0.0439 ±0.02440	0.0723 ±0.00263	0.0572 ±0.02374
**Lys**	0.0139 ±0.02318	0.0356 ±0.02159	0.0333 ±0.00376	0.0210 ±0.02386
**Met**	0.0032 ±0.00587	0.0055 ±0.00211	0.0174 ±0.00149	0.0050 ±0.00627
**Phe**	0.0334 ±0.02062	0.0367 ±0.02188	0.0344 ±0.00219	0.0343 ±0.01995
**Pro**	0.0157 ±0.03960	0.0039 ±0.01650	0.1174 ±0.00959	0.0213 ±0.04465
**Ser**	0.1455 ±0.15386	0.0660 ±0.02238	0.0558 ±0.00245	0.1178 ±0.13120
**Thr**	0.0595 ±0.01956	0.0538 ±0.01834	0.0440 ±0.00337	0.0567 ±0.01884
**Trp**	0.0005 ±0.00129	0.0023 ±0.00549	0.0080 ±0.00037	0.0016 ±0.00359
**Tyr**	0.0179 ±0.01621	0.0563 ±0.05206	0.0126 ±0.00177	0.0272 ±0.03355
**Val**	0.0583 ±0.02954	0.0487 ±0.01737	0.0605 ±0.00371	0.0560 ±0.02583

Environmental determinations of Asp/Asn and Glu/Gln did not distinguish between the two amino acids in the pairs, therefore they were considered together for the analysis.

**Figure 1:**
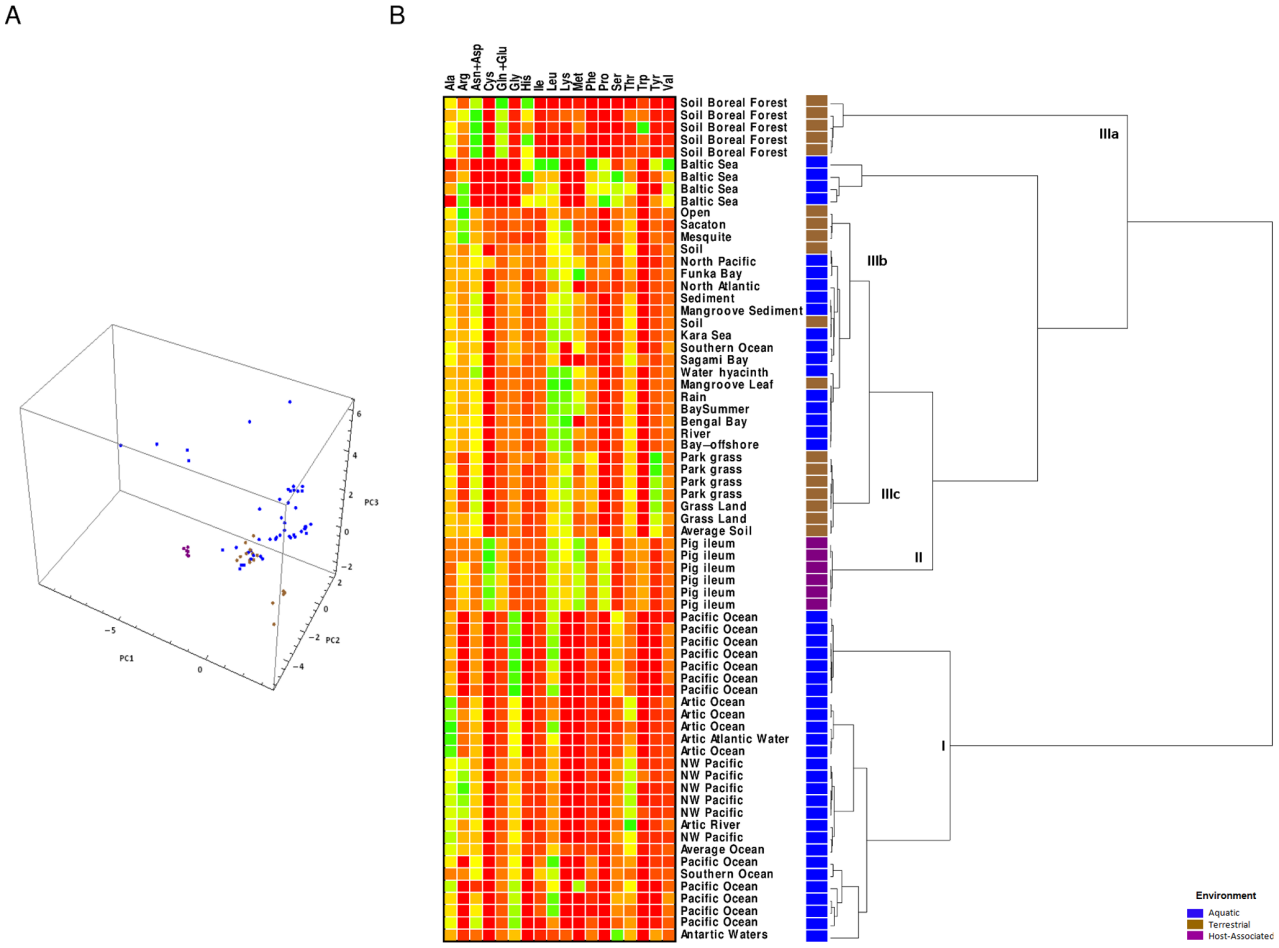
Characterization of different environments by their relative amino acid composition. A) scatter plot by Principal Component Analysis according to the type of environment; B) Hierarchical clustering analysis. The length of branches represents the degree of dissimilarity between clusters. The x-axis of the heat map represents the 20 amino acids by alphabetical order of the three-letter code name. Determinations of Asp/Asn and Glu/Gln were considered together for the analysis, because environmental measurements did not distinguish between the two amino acids in the pairs. The y- axis of the heatmap represents the individual environments where amino acid abundance was determined. Over- and under-representation of amino acid residues in each environment are represented in green and red colored squares, respectively.

PCA was performed on the multidimensional matrix of eRAAA for the environments. The principal components were used to investigate how environments grouped as a function of their amino acid composition. PCA showed segregation of water, soil and intestinal environments with respect to eRAAA, as observed in **Figure** **1A**. Eight principal components were needed to explain more than 90% of the variation in the environmental composition data (91.7%).

**Figure 1B** represents the clustering of environments on the basis of their eRAAA. Overall, similar environments clustered together. Environments were segregated in clusters corresponding to ocean waters, soil and host-associated environments, indicating the presence of habitat-specific trends in eRAAA. In general, ocean waters (**cluster I**) showed relative higher abundance of Ala, Trp, Gly, Gln+Glu and Leu. Host-associated environments (**cluster II**), showed relative higher abundance of Cys, Leu, Lys, Met and Pro. Terrestrial environments (**cluster III**) grouped into three sub-clusters: a) soil boreal forest, characterized by higher abundance of Asn+Asp, Gln+Glu, His and Ala; b) sandy soils (sacaton, mesquite, open) characterized by higher content of Arg, Lys, Leu, Ala and Asn+Asp and c) grass land environments, richer in Tyr, Lys, Asn+Asp, Ala, Leu and Thr.

Taken together these results suggest that the abundance of specific sets of amino acids creates signatures that are particular for each environment. However, it should be noted that Spearman rank correlations between the eRAAA of each pair of environments were statistically significant, ranging between 0.517 (*p-*value < 0.05) and 0.860 (*p-*value < 0.001). This indicates that, although eRAAA of different environments are specific to that environment and significantly different from those of other environments, the absolute differences between environments are small.

### Prediction of cellular amino acid abundance

To answer the third and fourth questions we estimated the cellular amino acid abundance of the organisms inhabiting the different environments, using CAI and δ (see methods) as predictors of an organisms' amino acid cellular abundance. These indexes weight the contribution of a given protein to the cellular amino acids pool by its predicted relative abundance with respect to ribosomal proteins. To validate this approach, predictions were compared with published cRAAA of reference organisms. Spearman rank correlations ranged from 0.743 to 0.861, showing that estimated amino acid abundances correlated highly significantly (*p*-values < 0.001) with experimental determinations (**Table** **2**). On average, higher correlations were obtained considering amino acid abundances weighted by δ. Nevertheless, CAI-weighted cellular amino acid abundances also highly and significantly correlated with the experimental determinations, whereas unweighted RAAA in the full proteomes of the test organisms correlated to the experimental determinations with significantly lower Spearman correlations (**Table** **2**).

**Table 2:** Spearman rank correlation coefficients between estimated amino acid compositions (based on CAI and δ predictors) and experimentally-determined amino acid abundances.

Organism	Description	Correlation	p-value^2^
	ƒaa^1^ *vs.* experimental data (Sauer *et al.*, 1996)	0.783	***
*Bacillus subtilis*	CAI *vs.* experimental data (Sauer *et al.*, 1996)	0.789	***
	δ *vs.* experimental data (Sauer *et al.*, 1996)	**0.812**	***
	ƒaa^1^ *vs.* experimental data (Pramanik & Keasling, 1998)	0.846	***
*Escherichia coli*	CAI *vs.* experimental data (Pramanik & Keasling, 1998)	0.837	***
	δ *vs.* experimental data (Pramanik & Keasling, 1998)	**0.857**	***
	ƒaa^1^ *vs.* experimental data (Okayasu *et al.*, 1997)	0.854	***
*Escherichia coli*	CAI *vs.* experimental data (Okayasu *et al.*, 1997)	0.847	***
	δ *vs.* experimental data (Okayasu *et al.*, 1997)	**0.861**	***
	ƒaa^1^ *vs.* experimental data (Okayasu *et al.*, 1997)	0.775	***
*Staphylococcus aureus*	CAI *vs.* experimental data (Okayasu *et al.*, 1997)	0.743	***
	δ *vs.* experimental data (Okayasu *et al.*, 1997)	**0.823**	***

Values in bold indicate the strongest correlation.

1 ƒaa indicates unweighted amino acid frequency in the complete predicted proteome of an organism.

2 *** *p*<0.001.

Further calculations and analysis were performed using both indexes δ and CAI and produced similar results. For convenience, data shown refers to the δ predictor only.

### Organisms did not segregate according to habitat or lifestyle

**Figure 2:**
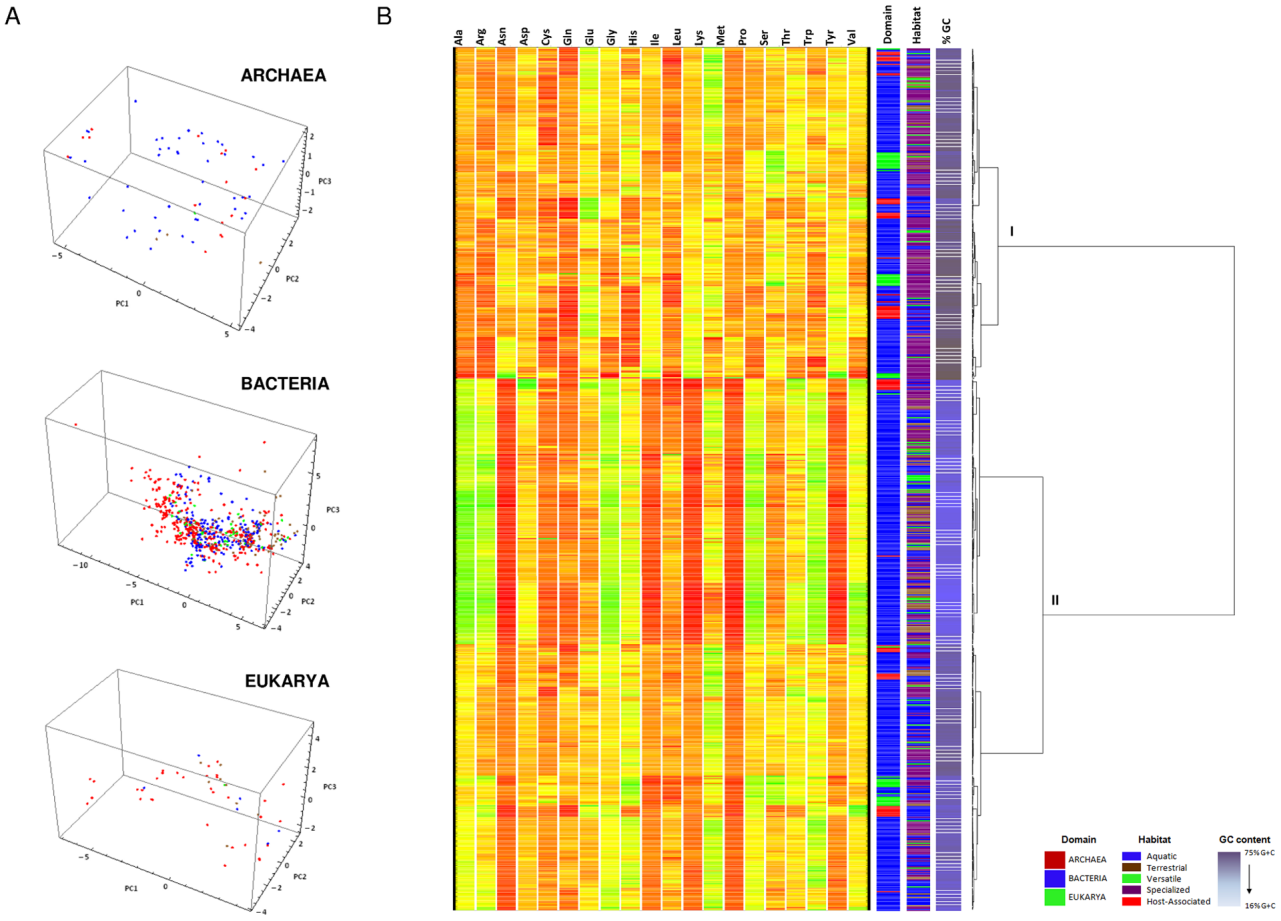
Characterization of the relative amino acid composition of the proteomes from different organisms. A) scatter plot by Principal Component Analysis according to the type of environment; B) Hierarchical clustering analysis. The length of branches represents the degree of dissimilarity between clusters. The x-axis of the heat map represents the 20 amino acids by alphabetical order of the three-letter code name. The y- axis of the heatmap represents the individual organisms where amino acid abundance was estimated. Over- and under-representation of amino acid residues in each organism are represented in green and red colored squares, respectively.

To investigate whether relative cellular abundance of amino acids also contained a signature of the environment in which the organisms have evolved, we performed PCA of the cRAAA and correlated each organism with its main environment. **Figure** **2A** shows the PCA analysis for the amino acid composition of organisms colored according to the type of habitat. Projection of the data in the 3 largest components accounted for 78.14%, 75.38% and 72.67% of the variation among organisms belonging to Archaea, Bacteria and Eukarya domains, respectively. However, no segregation of habitats by principal components was observed (**Figure** **2A**). Similar results were obtained considering lower taxonomic levels (phyla and classes) as well as when neglecting relative expression levels (data not shown).

Analyses based only on the amino acid composition of ribosomal proteins and RNA polymerases provided similar trends, with the 3 largest components accounting for only 62.77%, 62.70% and 66.14% of the variation among Archaea, Bacteria and Eukarya domains, respectively (**Figure** **S1**).

Hierarchical clustering analysis grouped organisms into two main clusters. The first cluster (**cluster I**) included organisms from the three domains and different habitats, which share relatively homogeneous amino acid abundance and GC content lower than 50%. The second cluster (**cluster II**) included organisms from the three domains and different habitats, but possessing higher content of Ala, Arg, Gly, His, Pro, Trp and Val, lower relative abundance of Asn, Ile, Lys, Pro, Tyr, and a GC content higher than 50% (**Figure** **2B**). Thus, segregation of amino acid relative compositions was not habitat- or domain-specific, being more constrained by GC content.

### %GC is the major factor influencing amino acid composition

To understand the contribution of the different factors on the cellular RAAA, we performed a more detailed analysis by modeling amino acid composition as a function of phylogeny, GC content and habitat using generalized linear models.

Best fit models obtained confirmed that in 17 out of 20 amino acids GC% was the major factor influencing amino acid composition. In Ala, Arg, Asn, Gly, Ile, Lys, Pro and Tyr, that effect was higher than 75% (**Table** **3**). Phylogeny was the factor that impacted most on Cys, Gln and Met relative abundance, although explaining only about 3% of the variance.

**Table 3:** Linear regression models for the effect of GC content, Phylogeny and Habitat on the relative cellular amino acid abundance.

Amino Acid	Variable	Function	Adjusted R^2^	p-value^1^
	%GC	12.3823+416.765x	0.888	***
Ala	Phylogeny	14.78−0.209605x	0.019	***
	Habitat	3.28+0.0541254x	0.030	***
	%GC	10.0666+708.622x	0.892	***
Arg	Phylogeny	14.78−0.0953123x	0.003	n.s.
	Habitat	3.28+0.0627061x	0.041	***
	%GC	77.0024−682.993x	0.815	***
Asn	Phylogeny	14.78+0.213086x	0.034	***
	Habitat	3.28+0.0618256x	0.038	***
	%GC	18.0057+587.185x	0.085	***
Asp	Phylogeny	14.78−0.0699774x	0.018	***
	Habitat	3.28+0.052295x	0.028	***
	%GC	53.5793−362.54x	0.009	***
Cys	Phylogeny	14.78−0.0835933x	0.028	***
	Habitat	3.28+0.0504854x	0.025	***
	%GC	50.0219−13.8308x	−0.001	***
Gln	Phylogeny	14.78−0.0940546x	0.036	***
	Habitat	3.28+0.0485648x	0.023	***
	%GC	79.8505−472.873x	0.134	***
Glu	Phylogeny	14.78−0.162344x	0.096	***
	Habitat	3.28+0.0319903x	0.009	***
	%GC	−16.8867+945.005x	0.839	***
Gly	Phylogeny	14.78+0.420635x	0.120	***
	Habitat	3.28+0.0704056x	0.046	***
	%GC	13.4426+1730.45x	0.230	***
His	Phylogeny	14.78−0.194095x	0.122	***
	Habitat	3.28+0.034075x	0.010	***
	%GC	86.7595−563.15x	0.849	***
Ile	Phylogeny	14.78−0.453057x	0.130	***
	Habitat	3.28+0.0538274x	0.026	***
	%GC	1.94247+470.982x	0.077	***
Leu	Phylogeny	14.78−0.11572x	0.051	***
	Habitat	3.28+0.0486466x	0.023	***
	%GC	76.1764−470.209x	0.866	***
Lys	Phylogeny	14.78−0.0723698x	0.002	n. s.
	Habitat	3.28+0.0618894x	0.040	***
	%GC	61.4524−479.803x	0.016	***
Met	Phylogeny	14.78−0.091508x	0.034	***
	Habitat	3.28+0.0488429x	0.024	***
	%GC	104.337−1332.69x	0.682	***
Phe	Phylogeny	14.78+0.13288x	0.022	***
	Habitat	3.28+0.0707417x	0.056	***
	%GC	−0.615608+1159.53x	0.841	***
Pro	Phylogeny	14.78−0.145668x	0.013	***
	Habitat	3.28+0.0642624x	0.043	***
	%GC	89.6811−655.588x	0.248	***
Ser	Phylogeny	14.78−0.00453643x	−0.001	n. s.
	Habitat	3.28+0.063256x	0.042	***
	%GC	15.6874+650.185x	0.089	***
Thr	Phylogeny	14.78−0.0849289x	0.027	***
	Habitat	3.28+0.0493972x	0.024	***
	%GC	7.78436+3584.66x	0.617	***
Trp	Phylogeny	14.78−0.15309x	0.037	***
	Habitat	3.28+0.0582441x	0.034	***
	%GC	93.3622−1407.74x	0.764	***
Tyr	Phylogeny	14.78−0.333038x	0.110	***
	Habitat	3.28+0.0519745x	0.025	***
	%GC	−11.348+863.332x	0.496	***
Val	Phylogeny	14.78+0.170741x	0.061	***
	Habitat	3.28+0.0768137x	0.058	***

1 *** *p*<0.001; n.s., not significant.

**Figure 3:**
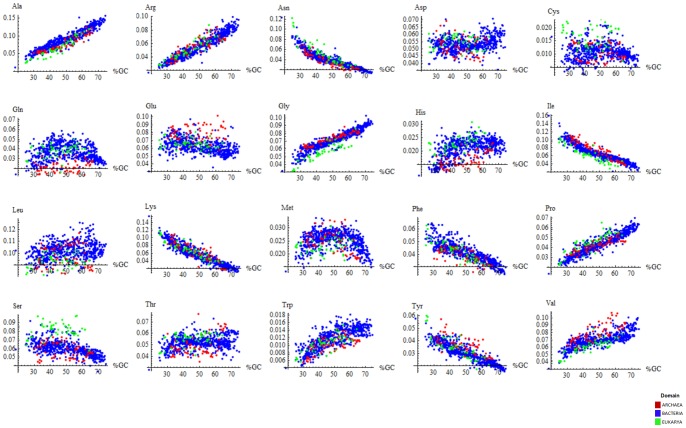
Relative amino acid composition, weighted by δ index, of each organism plotted against average GC content.

In fact, amino acid composition plotted against average GC content showed a strong correlation with the majority of amino acids, being Asp, Cys, Gln, Glu, His, Leu, Met, Ser, Thr the amino acids least affected by GC composition (**Figure** **3**). Results obtained considering the entire set of proteins in the genome did not differ from those obtained when considering only the set of highly expressed proteins (data not shown). The same trends were also observed considering amino acid compositions not weighted by expression.

**Figure 4:**
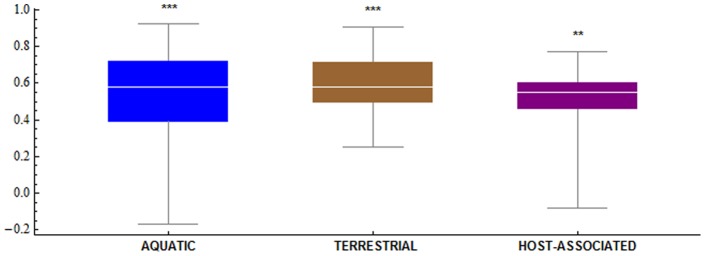
Spearman Rank Correlations between the RAAA of organisms and environments. Asterisks represent significance at *p*<0.01 (**) and *p*<0.001 (***).

Finally, we determined Spearman rank correlations between cellular and environmental RAAAs. Correlations were high and significant (**Figure** **4**), although the correlation between the cRAAA of a given organism and that of its environment was not significantly different from the correlation between the composition of the same organism and that of non-cognate environments for that organism.

Thus, our results suggest that the cRAAA are not unequivocally determined by the eRAAA in their habitats and that there is a complex and dynamic relationship between the relative amino acid abundance of an environment and that of its inhabiting organisms.

## Discussion

The ability to both adapt to and change its environment is a characteristic of life. Such changes are observed from macroscopic to microscopic and chemical scales. It is known that the relative amount of the major chemical elements that compose organisms is similar to that of environments. The accepted explanation for this is that, over time, cells have modified the environmental chemical landscape in such a way that it becomes more similar to themselves [1], [2].

To the best of our knowledge, whether a similar process is observed at the molecular level had not been analyzed before. In this work, we performed such an analysis by focusing on L-α-amino acids and their relative abundance in the environments and in cells. Given that L-α-amino acid production on Earth environments is only biological, one might expect that those abundances would be relatively constant. However, given that many microorganisms can efficiently scavenge amino acids from the environment one might also expect that the RAAA ratios are a dynamic result of the balance between amino acid release to the environment and amino acid uptake from the environment by the biota. Our results support that there is a relative abundance of the different amino acids in environmental source that is approximately constant.

Nevertheless, specific sets of amino acids were enriched in specific habitats, creating molecular signatures. The following environment-specific patterns of increased RAAA were observed: Ala, Trp, Gly, Gln+Glu and Leu, in oceans; Cys, Leu, Lys, Met and Pro, in host-associated environments; Asn+Asp, Gln+Glu, His and Ala, in soil boreal forest; Arg, Lys, Leu, Ala and Asn+Asp in sandy soils; and Tyr, Lys, Asn+Asp, Ala, Leu and Thr, in grass environments.

When it comes to identifying a direct correlation between environmental and cellular RAAA, our findings again suggest that such abundances correlate well globally. In contrast, when looking at how the cRAAA of specific organisms correlate to that of their cognate environments, we find that such correlations cannot be used to infer the environment from which the organism was extracted. These findings are apparently at odds with those from a previous study [11] that found both GC content and amino acid content of metagenomic datasets to be influenced by their environment. However, that study does not take into account environmental amino acid determinations and only four metagenomes are analyzed.

In fact, there is a large body of work on the compositional biases of genomes and proteomes and what controls such biases. Examples of such compositional biases and their probable causes are known at the nucleotide [29], [30], codon [31] and amino acid [9], [10], [32]–[35] levels. Nevertheless, such studies involved a limited number of sequences/organisms and only looked at the amino acid composition of the individual proteins, not that of the whole cells.

Our analyses took into account the relative amino acid compositions of proteins weighted by predicted levels of expression. These predictions were based on metrics that compare codon utilization between the ribosomal coding genes and other protein-coding genes [18]–[21]. More sophisticated estimations could also be achieved considering aa-tRNA abundance and ribosome occupancy as well [36]–[38]. However, aa-tRNA abundances are only well known for a small number of organisms and under very specific conditions. Estimations of aa-tRNA abundance based on the number of genes coding for tRNAs could also be used [39]. However, this could be biased by the varying quality of the genome annotation for each organism, given the size of the dataset used in this study.

Correlations between the cRAAA calculated using CAI and δ indexes and experimentally determined mRNA and protein abundance were not significant (data not shown). However, both CAI- and (more notably) δ-calculated cellular RAAA highly and significantly correlated with experimentally determined amino acid compositions. To the best of our knowledge, this constituted the first study on the relative amino acid compositions across domains, taking into account differential gene expression.

Hierarchical clustering analysis and PCA showed no apparent segregation of organisms according to habitat or domain. Clustering of amino acid relative content weighted by CAI and δ indexes showed segregation of organisms with higher content of residues with GC-rich codons (Ala, Arg, Gly, Pro) and organisms with higher content of residues with AU-rich codons (Asn, Ile, Lys, Phe, Tyr). A recent study [40] found similar results and reported that overall amino acid usage in Archaea is dominated by GC-bias. Lightfield and co-workers [41] also reported that distantly-related bacterial genomes with similar GC content have similar patterns of amino acid usage. Analyses of Sargasso's Sea shotgun sequencing reads have also shown an overrepresentation of AU-rich residues in such low-GC environments [11]. Taken together, these strongly suggest that amino acid composition of organisms cannot be directly predicted from their cognate environments and are strongly dependent on the GC content of their genomes.

Our generalized linear model analysis showed that, with the exception for Cys, Gln and Met, the variation in the cellular RAAA of all other amino acids was clearly explained by the variation in the GC content of the genomes. Given that the genomes of the organisms we are looking at are mostly constituted by gene coding sequences, such GC dependency could be a result of the relative abundance of amino acids coded by GC-rich (Ala, Gly, Pro, Arg and Ser) and/or GC-poor (Phe, Ile, Lys, Met, Asn, Tyr, and Leu) codons in the genes. However, the observation that the genomic GC content is the factor that explains the largest amount of variation in cRAAA for 17 out of 20 amino acids indicates that the dependency of cRAAA on genomic %GC content is not strongly affected by the GC content of codons.

The variation on the cRAAA of Cys, Gln and Met was, in contrast, influenced mainly by organism's phylogeny. Models based on mutation and selection in nearly 600 genomes, also suggest that GC content drives codon usage (and implicitly amino acid composition), rather than the reverse [42].

In conclusion, our findings are consistent with environmental amino acid abundances following relationships that are analogous to those of the Redfield ratios for chemical elements. Our results point to the existence of specific amino acid signatures that are particular for each environment, while also indicating that there are global relationships between the relative amino acid abundance in different environments. In contrast, the relative amino acid composition of organisms is not highly determined by the environment, even if the environmental composition is undoubtedly determined by its community of resident microorganisms. This is consistent with the existence of a complex and dynamic relationship between the RAAA of an environment and that of its inhabiting organisms, suggesting that individual organisms have evolved the capacity to mold their amino acid composition selectively, in a manner that is mostly independent from the eRAAA.

## Supporting Information

Figure S1 Principal Component Analysis of organisms as a function of cRAAA considering A) all predicted protein sequences in the genome [as shown in Figure 2A] and B) only ribosomal proteins and RNA polymerases.
(TIF)

Table S1 **Relative amino acid composition of different environments, as reported in and calculated from the literature.**
(XLSX)

Table S2 **Relative amino acid composition of different organisms calculated in this study based on predicted protein sequences of fully sequenced organisms (faa: relative amino acid frequency; δaa: relative amino acid frequency weighted by d index; CAIaa: relative amino acid frequency weighted by CAI index).**
(XLSX)
