# Supplementary figures and images for: Relative Amino Acid Composition Signatures of Organisms and Environments
Source: PLoS One. 2013 Oct 25;8(10):e77319. doi: 10.1371/journal.pone.0077319 (PMC3808408; doi:10.1371/journal.pone.0077319)

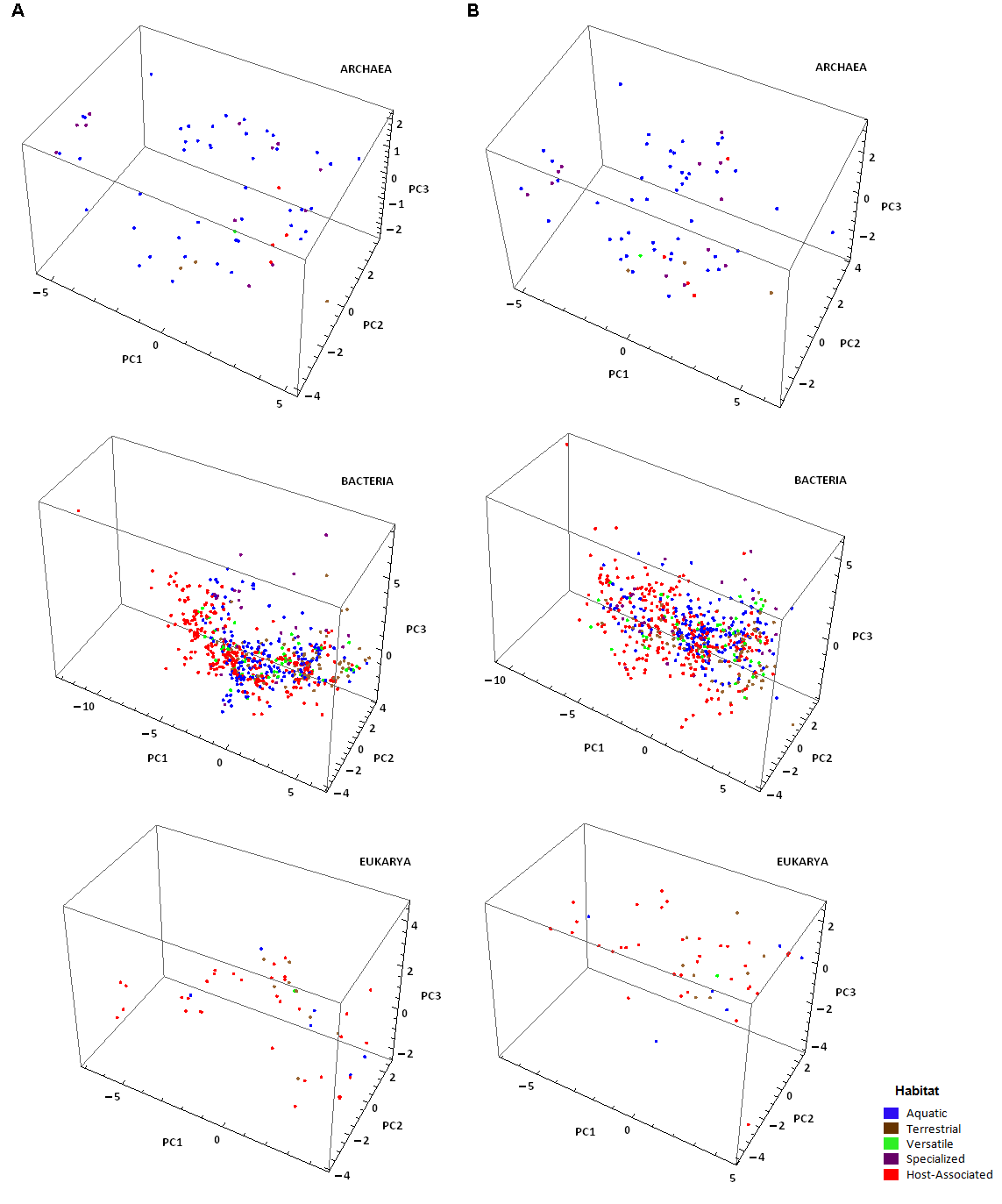

Supplement: Figure S1 — Principal Component Analysis of organisms as a function of cRAAA considering A) all predicted protein sequences in the genome [as shown in Figure 2A] and B) only ribosomal proteins and RNA polymerases. (TIF) [file pone.0077319.s001.tif]
